# Endometriosis Patients Show an Increased M2 Response in the Peritoneal CD14^+low^/CD68^+low^ Macrophage Subpopulation Coupled with an Increase in the T-helper 2 and T-regulatory Cells

**DOI:** 10.1007/s43032-020-00211-9

**Published:** 2020-06-22

**Authors:** Quanah J. Hudson, Kazem Ashjaei, Alexandra Perricos, Lorenz Kuessel, Heinrich Husslein, Rene Wenzl, Iveta Yotova

**Affiliations:** Department of Obstetrics and Gynecology, Medical University of Vienna, Waehringer Guertel 18-20, A-1090 Vienna, Austria

**Keywords:** Peritoneal immune microenvironment, Macrophage subtypes, T-regulatory cell, T-helper cell, Endometriosis, CD14, CD68, M1, M2

## Abstract

Endometriosis is a chronic inflammatory disease associated with an impaired immune response at the site of lesion implantation. The ability of macrophages to respond to changes in their environment is critical for an effective immune response. However, the existing knowledge of the peritoneal immune cell populations, their activation state and contribution to the immunological changes that occur in endometriosis are still controversial and inconclusive. In this study, we have examined the relative abundance of peritoneal macrophage subtypes, in women with (*n* = 21) versus without (*n* = 18) endometriosis and disease-associated changes in the adaptive T cell response. Using flow cytometry, we showed that peritoneal fluid monocyte/macrophages are composed of two populations of cells that exhibit major differences in the levels of the CD14 and CD68 markers, which we classified as the CD14^+low^/CD68^+low^ and CD14^+high^/CD68^+high^ subpopulations. Moreover, endometriosis-associated changes in the macrophage subtypes occurred only in the CD14^+low^/CD68^+low^ subpopulation. In this subpopulation, we found an increased macrophage type 2 response that was coupled with an increase in peritoneal T-helper 2 and T-regulatory cell populations in women with endometriosis, compared with controls. In summary, this study resolves conflicting data in the literature regarding changes in the peritoneal immune cell population in endometriosis and identifies CD14^+low^/CD68^+low^ macrophages as the subpopulation that changes in response to the disease.

**Electronic supplementary material:**

The online version of this article (10.1007/s43032-020-00211-9) contains supplementary material, which is available to authorized users.

## Introduction

Endometriosis is a common and often debilitating disease that affects 6–10% of women of reproductive age with prevalence rising to 35–50% in women with pelvic pain and infertility [1, 2]. The disease is characterized by the growth of endometrial lesions outside the uterus, most commonly on organs in the peritoneal cavity, with clinical symptoms that can include dysmenorrhea, dyspareunia, chronic pelvic pain and infertility [3, 4]. There are several hypotheses that explain the etiology of the disease. Among them, retrograde menstruation is the most widely accepted and is supported by the presence of viable endometrial cells in the menstrual reflux into the peritoneal cavity, indicating that they may implant and develop lesions that infiltrate the pelvic organs [5]. However, given that retrograde menstrual reflux occurs in most women, this hypothesis explains the source of endometrial cells but not the cause of the disease and the high level of heterogeneity in the symptoms. In order for endometriosis lesions to establish, they must evade the immune system; therefore, a defective immune response in the peritoneal cavity may be a critical factor in the establishment and progression of the disease [6].

Endometriosis is recognized as a chronic inflammatory disease associated with an impaired immune response at the site of lesion implantation [7]. The activation of an inflammatory response leads to local production of cytokines and chemokines that enhance the growth of the ectopic endometrial tissue both by inhibiting normal apoptotic processes and promoting local angiogenesis [8]. Therefore, research on understanding the role of the local immune environment in endometriosis has focused on the peritoneal immune cell population and soluble factors in the peritoneal fluid [9]. Deregulated T cell immunity [10] and suppressed function of NK cells in endometriosis [11] were found to contribute to an impaired immune response at the site of lesion growth. It has also been reported that women with endometriosis have an increased peritoneal fluid (PF) volume [12] and total number of peritoneal macrophages [13].

Macrophages belong to the mononuclear phagocyte system located throughout the body that is part of both the innate immune system and the adaptive immune system through their role as an antigen-presenting cell for T helper cells [14]. Macrophages in the peritoneum and elsewhere in the body can be broadly classified as classical pro-inflammatory (MΦ type 1) or alternately activated anti-inflammatory (MΦ type 2) subtypes [14, 15], although recent observations in mice indicate that a much wider spectrum of physically, functionally and developmentally distinct pMΦ subtypes can exist in the peritoneum [16, 17]. Studies in mouse indicate that peritoneal macrophages (pMΦ) are a mix of embryonic-derived tissue-resident macrophages and macrophages derived from monocytes originating in the bone marrow [18]. Depletion of pMΦ in an in vivo rat model of endometriosis led to a reduction of lesion implantation rate and size indicating that a defective peritoneal macrophage response may promote lesion development [19]. Several lines of evidence indicate that peritoneal macrophages in endometriosis may exhibit poor phagocytic ability [20], and that M2 macrophages can infiltrate endometriotic lesions and promote angiogenesis [21, 22]. Accumulated in vitro and in vivo studies demonstrate that an abnormal pMΦ response is strongly associated with the development of the disease [13, 21, 23]. However, the mechanisms responsible for the abnormal condition of pMΦ in endometriosis remain unknown. A recent report that the composition of pMΦ subtypes does not differ between women with and without the disease [13] contrasts with earlier reports that showed an increased frequency of the pMΦ2 subtype in an endometriosis mouse model [21] and in patients with endometriosis [21, 23]. It is critical to resolve these conflicting reports about the activation state of pMΦ in women with endometriosis in order to understand the role of pMΦ in the disease.

pMΦ participate in multiple aspects of innate and acquired immunity in the peritoneal cavity, and can release large quantities of pro-inflammatory and anti-inflammatory cytokines. This leads to the differentiation of CD4^+^ T cells into T helper (T_h_1, T_h_2, T_h_17) and T regulatory cells (T_reg_), thus playing a key role in stimulating the host immune response [24, 25]. Therefore, endometriosis-associated changes in innate immune response, and in particular at the pMΦ population of cells in women with endometriosis, could affect the frequency of T cell populations and their products (i.e. cytokines and chemokines) as part of adaptive immune responses.

Therefore, in this study, we aimed to resolve the conflicting reports on the activation state of peritoneal macrophages in endometriosis, and their relationship to T cells, and to identify and characterize any subpopulation of these immune cells that is changed in the disease.

## Material and Methods

### Study Population

Patients eligible for participation in the study were premenopausal women between 18 and 50 years of age who were undergoing laparoscopic surgery because of suspected endometriosis, unexplained pelvic pain, adnexal cysts, infertility work-up or uterine fibroids. To exclude the influence of comorbidities, which may have an effect on the immune cell response, a detailed clinical anamnesis was obtained for each study participant. The information was collected using a study-specific questionnaire designed by our certified endometriosis center and filled out by the patients prior to laparoscopic intervention. Patients were excluded from the study who were pregnant at the time, had any history of malignant disease, had any acute or chronic inflammation or infection, or had taken hormones for the last 3 months. The presence or absence of endometriosis was confirmed visually, by laparoscopic biopsy and histopathologic analysis. The endometriosis disease stage was classified according to the revised classification of the American Society of Reproductive Medicine (rASRM) [26]. Patients who did not show any endometriotic lesions at laparoscopic evaluation were included in the control group. The study was approved by the Medical University of Vienna’s Ethics Commission, and all patients gave their written, informed consent prior to inclusion in the study.

### Sample Collection

The peritoneal fluid (PF) was collected from participating women (*n* = 52) intraoperatively via aspiration. To minimize systemic blood contamination of the samples, collection was performed prior to laparoscopic lesion resection. Patients lacking PF (*n* = 9) and samples that showed severe peripheral blood contamination (*n* = 4) were excluded from the study population.

### Flow Cytometry (FACS)

For characterization of the populations of MΦ and T cells in PF of patients and controls by flow cytometry, the PF samples were first centrifuged at 400×*g* for 10 min at 4 °C. The cell pellets were washed twice with PBS (Gibco, MA, USA) supplemented with 2% FBS. The cells were counted and aliquoted to six aliquots of 5 × 10^5^ cells per tube for fluorochrome-labelled antibody staining. The staining of the cells for flow cytometry was performed according to the manufacturer’s protocol (BD) as follows. MΦ characterization was performed by simultaneous staining with antibodies for the surface markers CD14 (general monocyte/MΦ marker), CD80 (T cell ligand), CD86 (T cell ligand), CD163 (scavenger receptor) and CD206 (endocytic receptor), as well as with an antibody for detecting the intracellular CD68 antigen.

T cell characterization was performed by simultaneous staining with an antibody against the surface marker CD4 (cluster of differentiation 4) in combination with antibodies for the intracellular T-bet (T-box transcription factor), RoRγ (transcription factor), FOXP3 (Forkhead-box-protein P3) and GATA3 (transcription factor) proteins.

Both the macrophage and T cell characterizations used a mix of surface and intracellular markers, so the cells had to be fixed and permeabilized to enable the intracellular markers to enter the cells. After washing, 2.5–5 × 10^5^ cells per tube were resuspended in 50-μL fluorescence-activated cell sorting (FACS) buffer (BD) and incubated with the surface marker antibodies at 4 °C in the dark for 45 min. Cells were then washed twice with FACS buffer (centrifuged at 400×*g* for 10 min then resuspended) and then resuspended in 1-mL fixation/permeabilization buffer and incubated at 4 °C in the dark for 30–60 min. The cells were then centrifuged at 400×*g* for 10 min, and resuspended in 100-μL permeabilization buffer (BD) together with the antibodies for the intracellular markers and incubated in the dark at room temperature for 45 min. Finally, cells were centrifuged at 400×*g* for 10 min and resuspended in 300-μL FACS buffer and run on the flow cytometer. Unstained cells and specific antibody isotype controls were used to control for each measurement. Non-viable cells were identified and excluded from the analysis using DAPI staining.

The fluorochrome-labelled antibodies used in this study are listed in Supplementary Table 1. Stained samples were detected on a BD FacsVerse flow cytometer (BD Biosciences, San Jose, CA, USA) with FACSuite v.1.0.5.3841 software (BD Biosciences, San Jose, CA, USA) and analyzed using FlowJo v.10.0.7 (BD Biosciences, San Jose, CA, USA). For each stain, 10,000 events per sample were counted.

### Statistical Data Analysis

All statistical tests were performed using SPSS version 17.0 for patient cohort characterization and Prism (GraphPad software, La Jolla, CA, USA) for the remaining experimental settings. The exact statistical procedures for each analysis is described in the corresponding figure legend.

## Results

### Patient Characteristics

Of the 39 women included in this study, 21 had endometriosis and 18 had no evidence of endometriosis and represented the control group. Patient characteristics are shown in Table 1. Of the women with endometriosis, 50% were classified as having minimal to mild (rASRM I, II) and 50% as having moderate to severe (rASRM III, IV) endometriosis. Of these patients, 28.6% had peritoneal and 7.1% had ovarian endometriosis, while 21% had lesions at multiple sites. In the control group, 8 women had ovarian cysts and 5 uterine fibroids, and five did not show any endometrial abnormalities.

**Table 1:** Baseline cohort characteristics.

	EM-free control (^b^)	EM (^b^)	*p*-value
number of patients	*n*=18	*n*=21	
Age	34.06 ± 8.11^a^	32.71 ± 8.17^a^	NS
BMI	25.04 ± 7.10^a^	24.8 ± 4.59^a^	NS
overall pain score	5.07 ± 3.77^a^(^1^)	7.62 ± 2.22^a^(^3^)	NS
parity	1.41 ± 1.46^a^(^1^)	0.76 ± 1.18^a^	NS
gravity	1.00 ± 0.87^a^ (^1^)	0.34 ± 0.65	NS
parity >0, n (%)	8 (47.1)	7 (33.3)	
gravity>0, n (%)	8 (47.1)	5 (23.8)	
rASRM stage of EM, n (%)			
I, minimal		7 (33.3)	
II, mild		3 (14.3)	
III, moderate		8 (38.1)	
IV, severe		3 (14.3)	
Lesion entity, n (%)			
ovarian EM		3 (14.3)	
peritoneal EM		9 (42.9)	
DIE		0	
Adenomyosis		0	
multi-location EM		9 (42.9)	

EM-endometriosis, BMI- body mass index, ns - not significant

^a^the values are given as a mean ± standard deviation. The differences between the groups were determined using the Kruskal-Wallis test

^b^number of missing values

### Peritoneal Macrophages Are Divided into CD14^+low^/CD68^+low^ and CD14^+high^/CD68^+high^ Subpopulations That Show Different Levels of the MΦ1 and MΦ2 Subtypes

In order to identify any changes that may occur in the pMΦ population in endometriosis, we sought to first characterize the state seen in both women with and without the disease. We isolated cells from the PF and stained for CD14 (LPS co-receptor), CD68 (scavenger receptor), CD80 (T cell ligand), CD86 (T cell ligand), CD163 (scavenger receptor) and CD206 (endocytic receptor). In humans, the lipopolysaccharide (LPS) receptor component CD14 and the lysosome-associated membrane glycoprotein (LAMP) CD68 are co-expressed in monocytes and macrophages, but not in other immune cell types, enabling them to be used together to identify these cells [27]. CD14 and CD68 can then be used in conjunction with CD80, CD86, CD163 and CD206 to identify specific subtypes of macrophages [27].

Following staining, the cells were subjected to flow cytometry analysis and gated according to size based on FSC-A (forward scatter area) vs. SSC-A (side scatter area), and then single cells were selected based on FSC-A vs. FSC-H (forward scatter height), with dead cells being excluded based on DAPI staining.

**Fig. 1:**
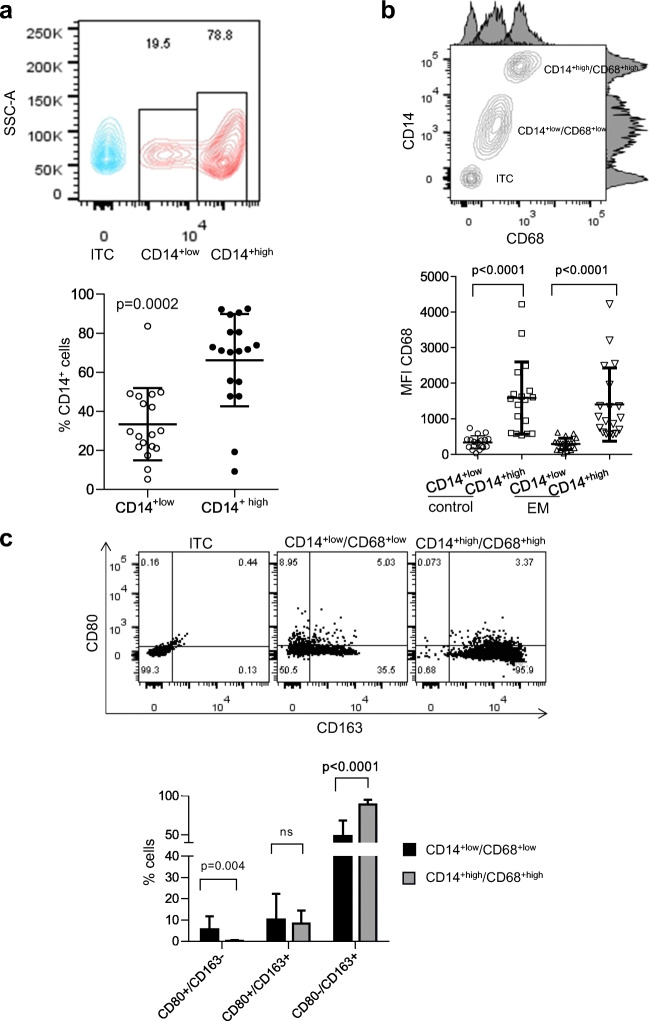
Peritoneal macrophages in women without endometriosis are divided into CD14^+low^/CD68^+low^ and CD14^+high^/CD68^+high^ subpopulations that differ in the levels of the MΦ1 and MΦ2 subtypes. **a** Flow cytometry analysis of peritoneal monocytes/macrophages (pMO/pMΦ) reveals CD14^+low^ and CD14^+high^ subpopulations. Top: a representative contour plot of CD14 versus side scatter analysis (SSC-A) shows the low and high CD14 subpopulations and the isotype control (ITC). Bottom: the CD14^+high^ subpopulation is significantly more abundant than the CD14^+low^ subpopulation. The data is presented as a bee swarm dot plot with the mean ± SD indicated, for a cohort of *n* = 18 women without endometriosis. Statistical analysis was conducted using a multiple *T* test with the Holm-Sidak method, using alpha = 0.05, for correction. Significant differences are indicated by the *p* value on the top of the graph. **b** CD14 and CD68 expression is correlated in pMΦ, with the CD14^+high^ subpopulation showing higher levels of CD68 expression than the CD14^+low^ subpopulation. Top: a representative contour plot of CD68 versus CD14 signal with histograms of CD68 and CD14 signal shows distinct CD14^+low^/CD68^+low^ and CD14^+high^/CD68^+high^ subpopulations in women without endometriosis (ITC, isotype control). Bottom: in both control patients and women with endometriosis, the expression level (mean fluorescence intensity, MFI) of CD68 is significantly higher in the CD14^+high^ than in the CD14^+low^ subpopulation. The data is presented as a bee swarm dot plot with mean values ± SD for *n* = 18 controls and *n* = 21 women with endometriosis. Statistical analysis was conducted using the Kruskal-Wallis test followed by Dunn’s multiple comparisons test. Significant differences are indicated by the *p* value on the top of the graph. **c** In women without endometriosis, the CD14^+low^/CD68^+low^ pMΦ subpopulation shows higher levels of the MΦ1 subtype and lower levels of the MΦ2 subtype than in the CD14^+high^/CD68^+high^ pMΦ subpopulation, while levels of the mixed MΦ1/MΦ2 subtype do not differ. Top: representative scatter plots showing the expression of the M1 marker CD80 vs. the M2 marker CD163 in the CD14^+low^/CD68^+low^ (middle) and CD14^+high^/CD68^+high^ (right) pMΦ subpopulations (isotype control, ITC; left). Bottom: The CD14^+low^/CD68^+low^ pMΦ subpopulation has significantly higher levels of the MΦ1 subtype and significantly lower levels of the MΦ2 subtype compared with the CD14^+high^/CD68^+high^ subpopulation, while levels of the mixed MΦ1/MΦ2 subtype do not significantly differ between the subpopulations. The bar graphs show the mean ± SD for each MΦ subtype. Statistical analysis was done using two-way ANOVA with Sidak’s multiple comparisons tests. Significant differences between the groups are indicated by the *p* value on the top of the graph

We first analyzed expression of CD14, a monocyte and macrophage marker [28]. We compared CD14^+^ cells to SSC-A revealing a CD14^+low^ and CD14^+high^ subpopulation that was present in the PF of both women with and without endometriosis (Fig. 1a, top and Supplementary Fig. 1a, left). In both women with and without endometriosis, the relative abundance of CD14^+low^ cells was significantly lower compared with that of CD14^+high^ cells (2.5 times for controls, *p* = 0.0002 and 1.7 times for endometriosis, *p* = 0.0005 respectively, Fig. 1a, bottom and Supplementary Fig. 1a, middle). As CD14 can be expressed in other immune cells, we then used a second marker, CD68, to specifically identify the peritoneal monocyte and macrophage populations [27, 29]. The expression level of CD14 and CD68 was correlated, with contour plots revealing distinct CD14^+low^/CD68^+low^ and CD14^+high^/CD68^+high^ subpopulations in both women with and without endometriosis (Fig. 1b, top and Supplementary Fig. 1a, right). This was confirmed by cells in the CD14^+high^ subpopulation showing a significantly higher CD68 mean fluorescence intensity (MFI) than cells in the CD14^+low^ subpopulation for both women with and without endometriosis, indicating that CD14 and CD68 levels are highly correlated in pMΦ (Fig. 1b, bottom). Therefore, the two different peritoneal monocyte/macrophage subpopulations were labelled CD14^+low^/CD68^+low^ and CD14^+high^/CD68^+high^.

We next determined the relative abundance of classical MΦ1 (CD68^+^/CD80^+^/CD163^−^) and alternatively activated MΦ2 (CD68^+^/CD80^−^/CD163^+^) macrophages in the CD14^+low^/CD68^+low^ and CD14^+high^/CD68^+high^ subpopulations of cells from patients and controls (Fig. 1c, top and Supplementary Fig. 1b, top). This analysis specifically identified macrophages from the CD14^+^/CD68^+^ cells as either CD80^+^ and/or CD163^+^, implying that the remaining CD80^−^/CD163^−^ cells were monocytes. This indicates that around 50% of the CD14^+low^/CD68^+low^ subpopulation and > 99% of the CD14^+high^/CD68^+high^ subpopulation were macrophages. The percentage of MΦ1- and MΦ2-positive cells differed significantly between the CD14^+low^/CD68^+low^ and CD14^+high^/CD68^+high^ pMΦ subtypes for both patients and controls. The CD14^+low^/CD68^+low^ subpopulation showed a higher proportion of MΦ1 cells (8.6 times, *p* = 0.0042 for controls and 5.35 times, *p* = 0.005 for endometriosis) and a lower proportion of MΦ2 cells (1.8 times, *p* < 0.0001 for both controls and 2.0 times, *p* < 0.0001 for endometriosis) (Fig. 1c, bottom and Supplementary Fig. 1b, bottom). Interestingly, in addition to MΦ1 and MΦ2 macrophages, we also identified a mixed MΦ1/MΦ2 subtype that was both CD80 and CD163 positive and present in the PF of both women with and without endometriosis (Fig. 1c, top and Supplementary Fig. 1b, top). However, the relative abundance of this mixed MΦ1/MΦ2 subtype did not significantly differ between the CD14^+low^/CD68^+low^ and CD14^+high^/CD68^+high^ subpopulations of macrophages in either women with or without endometriosis (Fig. 1c, bottom and Supplementary Fig. 1b, bottom).

Changes in the MΦ2 levels between the CD14^+low^/CD68^+low^ and CD14^+high^/CD68^+high^ subpopulations could be explained by difference in MΦ2 subtypes. Therefore, we examined the distribution and abundance of the MΦ2a (CD80^−^/CD163^+^/CD206^+^) and MΦ2b (CD80^−^/CD163^+^/CD86^+^) subtypes in controls and endometriosis in the CD14^+low^/CD68^+low^ and CD14^+high^/CD68^+high^ subpopulations (Supplementary Fig. 2a). However, there was no significant difference in the levels of the MΦ2a or MΦ2b subtypes, or the intermediate (CD80^+^/CD163^+^/CD86^+^) subtype, between the CD14^+low^/CD68^+low^ and CD14^+high^/CD68^+high^ subpopulations for either the controls or endometriosis patients (Supplementary Fig. 2b).

In summary, we identified two main subsets of peritoneal macrophages in this study: a CD14^+low^/CD68^+low^ subpopulation and a more abundant CD14^+high^/CD68^+high^ subpopulation. The CD14^+low^/CD68^+low^ subpopulation contained a higher proportion of classically activated MΦ1 cells and a lower proportion of alternatively activated MΦ2 cells than the CD14^+high^/CD68^+high^ subpopulation. We also identified a mixed MΦ1/MΦ2 subtype, but this did not differ between the CD14^+low^/CD68^+low^ and CD14^+high^/CD68^+high^ subpopulations. These relative differences between the macrophage subpopulations that we identified were similar in women with and without endometriosis.

### Peritoneal M1 Macrophages Decreased and M2 Macrophages Increased in Endometriosis in the CD14^+low^/CD68^+low^ Subpopulation

**Fig. 2:**
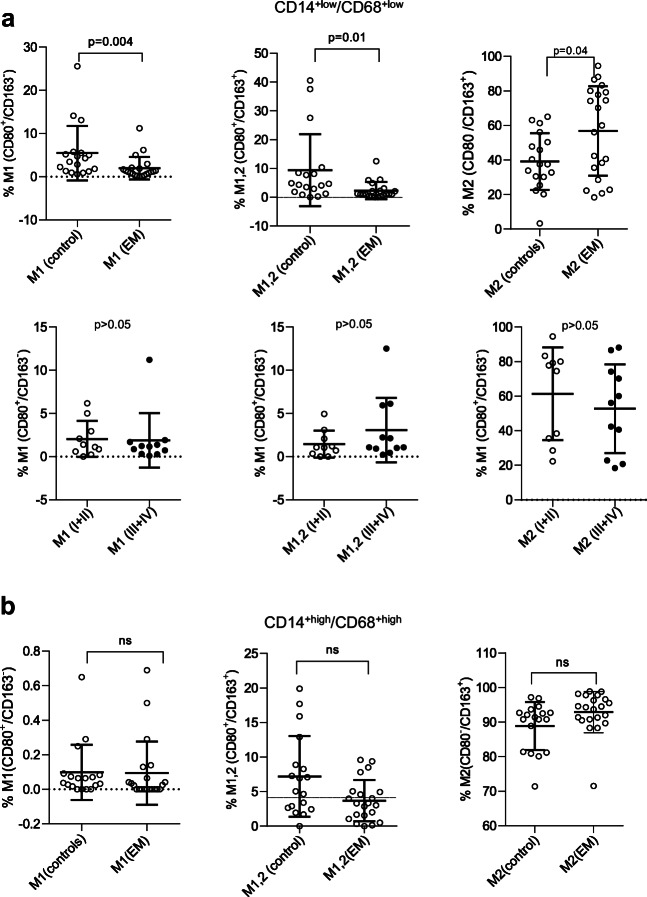
The MΦ1 and MΦ1/MΦ2 peritoneal macrophage subtypes decreased and the M2Φ subtype increased in the CD14^+low^/CD68^+low^ subpopulation in endometriosis. **a** Top: In the CD14^+low^/CD68^+low^ pMΦ subpopulation, endometriosis patients show a significant reduction in the MΦ1 (CD80^+^/CD163^−^, left) and MΦ1/MΦ2 (CD80^+^/CD163^+^, middle) subtypes, and a significant increase in the MΦ2 (CD80^−^/CD163^+^, right) subtype compared with controls. Bottom: No significant difference is seen in the MΦ1 (left), MΦ1/MΦ2 (middle) or MΦ2 (right) subtypes between patients with minimal and mild (rASRM stages I+II), compared with severe (rASRM stages III+IV) endometriosis in the CD14^+low^/CD68^+low^ pMΦ subpopulation. **b** In the CD14^+high^/CD68^+high^ pMΦ subpopulation, endometriosis patients show no significant differences to controls for the MΦ1 (left), MΦ1/MΦ2 (middle) and MΦ2 (right) subtypes. In **a** and **b**, each subtype is plotted as a percentage of the total cell number with distribution presented as a bee swarm dot plot with mean value ± SD for each group. Statistical analysis was conducted using multiple *T* tests with Holm-Sidak correction with alpha = 0.05. Significant differences are indicated by the *p* value on the top of each graph, non-significant differences by ns. Analysis was conducted on pMΦ from *n* = 18 controls and *n* = 21 endometriosis patients

Having characterized the pMΦ populations present in women with and without endometriosis, we next directly compared the subpopulations to identify any differences that exist in the disease state. We first compared the relative abundance of MΦ1, MΦ1/MΦ2 and MΦ2 macrophages present in the CD14^+low^/CD68^+low^ subpopulation in women with and without endometriosis. We found that both the MΦ1 (CD80^+^/CD163^−^) and mixed MΦ1/MΦ2 (CD80^+^/CD163^+^) subtypes were significantly decreased in women with endometriosis, while the MΦ2 (CD80^−^/CD163^+^) subtype was significantly increased (Fig. 2a, upper panel). However, these differences were not associated with the stage of the disease (Fig. 2a, lower panel). The age of the patient also showed no correlation with the changes in the composition of the macrophage population (Spearman’s correlation, *p* > 0.05). In the CD14^+high^/CD68^+high^ subpopulation, no statistically significant differences were observed (Fig. 2b), although we cannot exclude that a small non-significant increase seen in the M2 subtype may become significant with a larger sample size.

### T_h_2 and T_reg_ Cell Populations Are Increased in the Peritoneal Fluid of Women with Endometriosis

**Fig. 3:**
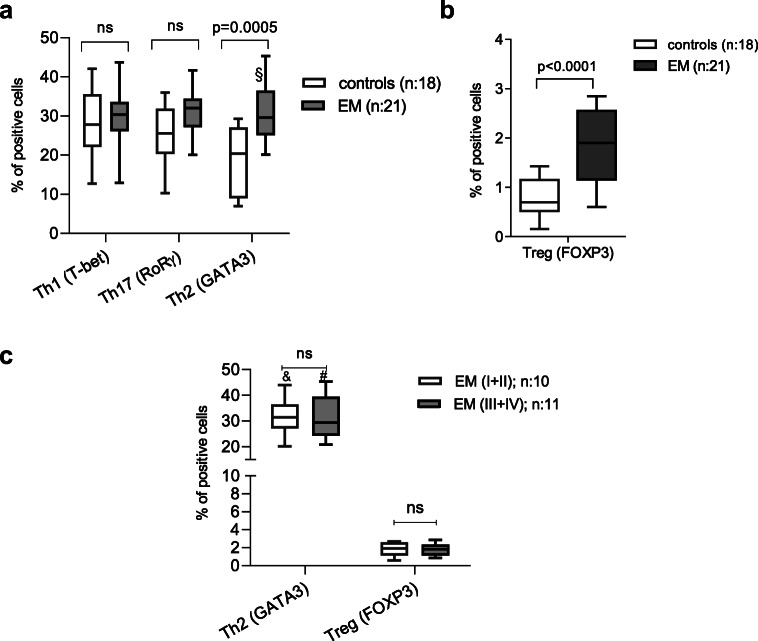
T_h_2 and T_reg_ cell populations are increased in the peritoneal fluid of women with endometriosis. **a** T_h_2 cells (CD4^+^/GATA3^+^) are significantly increased in the peritoneal fluid of women with endometriosis, while T_h_1 (CD4^+^/T-bet^+^) and T_h_17 (CD4^+^/RoRγ^+^) do not significantly differ compared with controls. Data are presented as box and whisker plots ranging from minimum to maximum, including the median and box boundaries at the 25th and 75th percentiles. As the sample sizes were not equal, these data were analyzed by fitting a mixed model, rather than by repeated measures ANOVA (which requires equal sample sizes). The significant differences between the groups are indicated by the *p* value on the top of the graph. *p* values < 0.05 are considered significant and non-significant differences are indicated by ns. Analysis was conducted on *n* = 18 controls and *n* = 21 endometriosis patients, except for T_h_2 where data for 3 endometriosis patients was missing (indicated by “§”). **b** T_reg_ cells (CD4^+^/FOXP3^+^) are significantly increased in the peritoneal fluid of women with endometriosis. Data are presented as box and whisker plots, ranging from minimum to maximum, including the median and box boundaries at the 25th and 75th percentiles. Statistical analysis was conducted using multiple *T* tests with Holm-Sidak correction with alpha = 0.05. Significant differences are indicated by the *p* value on the top of the graph. Analysis was conducted on *n* = 18 controls and *n* = 21 endometriosis patients. **c** The relative abundance of T_h_2 cells (CD4^+^/GATA3^+^) and T_reg_ cells (CD4^+^/FOXP3^+^) did not differ between women with minimal and mild (I+II) and severe (III+IV) endometriosis. Data are presented as box and whisker plots ranging from minimum to maximum, including the median and box boundaries at the 25th and 75th percentiles. As the sample sizes were not equal, these data were analyzed by fitting a mixed model, rather than by repeated measures ANOVA (which requires equal sample sizes). The significant differences between the groups are indicated by the *p* value on the top of the graph and non-significant differences are indicated by ns. Analysis was conducted on *n* = 18 controls and *n* = 21 endometriosis patients. “&” indicates missing values for T_h_2 (GATA3) in one patient with stage rASRM I; “#” indicates missing values for Th2 (GATA3) in two patients with rASRM III endometriosis

The secretion of cytokines and chemokines by pMΦ perpetuates the inflammatory response by recruiting additional innate immune cells, such as monocytes and neutrophils, and by inducing T cell differentiation [30, 31]. Although dendritic cells are classically considered to be the major drivers of CD4^+^ T_helper_ (T_h_) cell polarization, evidence is accumulating that macrophages can also play a role in this process [32]. Therefore, given this relationship between MΦ and T_h_ cells, we next assayed the relative abundance in PF of different T_h_ cell subtypes in women with and without endometriosis to determine if there is any correlation with the differences in the MΦ subpopulations. We found that in women with endometriosis, the relative abundance of T_h_2 (CD4^+^/GATA3^+^) cells was significantly higher (1.7 times, *p* = 0.0005) than in controls, whereas the levels of T_h_1 (CD4+/T-bet+) cells and T_h_17 (CD4^+^/Rorγ^+^) cells did not differ between patients and controls (Fig. 3a).

In addition to their effects on T_h_ cell polarization, activated macrophages can also positively or negatively influence the function of T_reg_ (CD4^+^/FOXP3^+^) cells through the production of soluble mediators such as TNFα and IL-6 [33, 34]. Therefore, we analyzed the relative abundance of T_reg_ (CD4^+^/FOXP3^+^) cells in the PF of women with and without endometriosis. We found that the relative abundance of T_reg_ cells is increased (2.3 times, *p* < 0.0001) in the PF of women with endometriosis (Fig. 3b). The increased T_h_2 and T_reg_ cell levels that we observed in endometriosis patients were not related to the stage of the disease (Fig. 3c).

## Discussion

In this study, we investigated whether endometriosis-associated differences in the peritoneal fluid monocyte/macrophage subpopulations of cells exist, and whether these are correlated with changes in the T cell immune response in women with the disease. We found that peritoneal fluid macrophages are composed of two populations of cells exhibiting major differences in the levels of expression of CD14 and CD68 markers, which we classified as the CD14^+low^/CD68^+low^ and CD14^+high^/CD68^+high^ subpopulations. Our research also revealed that endometriosis-associated changes in macrophage subpopulations occurred only in the CD14^+low^/CD68^+low^ population, where we found an increased MΦ2 response coupled with an increase in the peritoneal T_h_2 and T_reg_ cell populations in women with endometriosis, compared with controls.

Previous studies of peritoneal macrophages in endometriosis have focused on general populations of classical and alternatively activated cells [15]. These studies did not take into account the recent observations in mice [16, 17] and humans [35] that show the existence of physically, functionally and developmentally distinct peritoneal macrophage subsets. Using a mouse model of endometriosis, Yuan and colleagues [36] showed that pMΦ are composed of two distinct subpopulations: large peritoneal macrophages (LPM) and small peritoneal macrophages (SPM), which have a different origin and play distinct roles in the pathogenesis of the disease. The LPM are derived from embryonic tissue and play a crucial role in the early survival of refluent endometrial tissues, while SPMs are derived from peritoneal monocytes, and may be involved in the pathogenesis of endometriosis [36]. Interestingly, we also found two pMΦ subpopulations in humans that show a different functional response in endometriosis. Similar to the mouse, our data indicates that one pMΦ subpopulation, the CD14^+low^/CD68^+low^ cells, behaves abnormally in women with endometriosis, which may contribute to an impaired immune response after the initial development of endometriosis lesions. However, whether the human CD14^+low^/CD68^+low^ and CD14^+high^/CD68^+high^ subpopulations of pMΦ are the phenotypic and functional equivalents of the mouse LPM and SMP remains to be tested.

Previous reports have also subdivided peritoneal monocyte/macrophage cells using levels of CD14 expression. For example, Ruiz-Alcaraz et al. defined different peritoneal monocyte/macrophage subpopulations based on CD14 and CD16 expression levels [35]. In our study, around 50% of the CD14^+low^/CD68^+low^ subpopulation were negative for macrophage subtype markers CD80 and CD163, compared with less than 1% of the CD14^+high^/CD68^+high^ subpopulation. This indicates that the CD14^+low^/CD68^+low^ subpopulation may originate from monocytes in the circulation that then differentiate into macrophages, whereas the CD14^+high^/CD68^+high^ subpopulation may be tissue-resident macrophages. In support of this, Ruiz et al. also found a CD14^+high^ subpopulation, which they found expressed additional markers that indicated they were tissue-resident macrophages [35]. However, further evidence is required to verify the origin of the CD14^+low^/CD68^+low^ and CD14^+high^/CD68^+high^ subpopulations that we found in this study.

The literature regarding the activation state of pMΦ in women with endometriosis is contradictory. Most recently, it has been reported that the relative abundance of MΦ2 cells in the pMΦ population does not differ between women with and without the disease [13]. In contrast, earlier studies reported an increased frequency of peritoneal MΦ differentiated into MΦ2 type in an endometriosis mouse model [21] and in patients with endometriosis [21, 23]. Our study confirms and further extends the observations of these earlier studies, showing that the increase in the relative abundance of the MΦ2 subtype and the corresponding decrease of the MΦ1 subtype in endometriosis are limited to the CD14^+low^/CD68^+low^ pMΦ subpopulation. Additionally, we identify a mixed MΦ1/MΦ2 subtype that is only decreased in the CD14^+low^/CD68^+low^ pMΦ subpopulation in endometriosis. So-called chimeric macrophages with a mixed MΦ1/MΦ2 phenotype can have an impaired inflammatory function and have been described in inflammatory conditions, such as cancer [37], multiple sclerosis [38] and rheumatoid arthritis [39]. However, whether these cells have a similar phenotype and role in the pathogenesis of endometriosis remains to be investigated.

Coordinated regulation of the activation status of innate (MΦ) and adaptive (T cell) immune cells ensures an adequate immune response to changes in the peritoneal microenvironment [40, 41]. Polarization to a MΦ1 state arises in response to interferon-γ that is produced during adaptive immune response by T_h_1 cells, while MΦ1-secreted chemokines and cytokines such as CXCL9, CXCL10 and IL-12 can direct the T cell polarization to T_h_1 type cells [41]. Alternatively, MΦ2 cells are involved in coordinating a type II immune response together with T_h_2 and T_reg_ cells [32, 42]. In this study, we found that an increase in the relative abundance of pMΦ2 in endometriosis is accompanied by an increase in the relative abundance of T_h_2 type and T_reg_ cells. Such a shift towards a type II immune anti-inflammatory response may be a mechanism how endometriosis lesions become established [43, 44]. Recently, subfertility and ectopic lesion growth were also attributed to deregulation of T_reg_ cell levels. Higher abundance of these cells has been reported within ectopic lesions of murine model of endometriosis [45] and in eutopic tissues [46] and PF of women with versus without the disease [47]. Endometriosis patients, who have a higher chance of fertility problems, maintain a relatively constant high level of T_reg_ cells, whereas patients without endometriosis and with normal fertility have lower levels that vary throughout the menstrual cycle, indicating there may be a link to fertility [46]. Previous studies found an increase in the relative abundance of the T_h_17 population that was associated with an increased inflammatory response and increased severity of the disease in women with endometriosis [48–50]. In contrast, we found no significant difference in the relative abundance of peritoneal T_h_17 cells in women with and without endometriosis. However, as the median level of T_h_17 cells was increased in endometriosis, it is possible that variability in our relatively small study cohort did not allow a significant difference to be detected.

Ectopic endometriosis lesions that develop in the peritoneal cavity are exposed to the complex immune microenvironment of the peritoneal fluid that may influence their development. Conversely, the lesions secrete factors into the peritoneal microenvironment that may affect the immune cell response and immune cell differentiation and plasticity. Therefore, it can be difficult to dissect the effect of the lesions on the immune cells and vice versa. In general, it has been shown that the phenotype of polarized MΦ1 and MΦ2 macrophages can, to some extent, be reversed in vitro [51] and in vivo [52]. For example, in a peritoneal model of inflammation, resolution phase macrophages were shown to express a unique mixed MΦ1/MΦ2 phenotype, which could be repolarized to MΦ1 by changes in cAMP levels [53]. Here, we have shown that endometriosis is associated with changes in macrophage activation, with a shift from a MΦ1 to a MΦ2 type of immune response in the CD14^+low^/CD68^+low^ subpopulation, indicating some plasticity of the cells in the development of the disease.

Given that our results and those of others indicate the importance of the pMΦ phenotype in the pathology of endometriosis, approaches that influence the polarization state of pMΦ may have therapeutic potential in the disease. Recently, a novel mode of communication between endometriosis cells and macrophages has been uncovered. In an in vitro mouse macrophage model, exosomes isolated from primary stromal cells derived from the eutopic endometrium of endometriosis mouse model promoted polarization to an MΦ2 phenotype that showed reduced phagocytic ability, whereas control exosomes derived from normal endometrial stromal cells did not affect macrophage polarization [54]. Endometriosis-derived exosomes also promoted polarization of pMΦ to a MΦ2 phenotype following peritoneal injection and increased the growth of lesions in an endometriosis mouse model. Although these findings would have to be confirmed in humans, this indicates that targeting the release or modifying the transformative message of endometriosis exosomes might restore the MΦ1/MΦ2 balance and serve as a new therapy for the disease. An alternative approach may be to target specific subpopulations of pMΦ using so called prodrug-fluorophore conjugates to deplete macrophage subtypes to recover the balance of macrophage subtypes to treat the disease [55]. Both these approaches and others aiming to alter the balance of pMΦ subtypes as a therapy for endometriosis require the subpopulations in controls and endometriosis patients to be clearly defined. In this study, we have identified the CD14^+low^/CD68^+low^ pMΦ subpopulation as that that undergoes changes in MΦ polarization in endometriosis, but further characterization of this subpopulation will be required before such targeted therapies could be trialled as a treatment for the disease.

A general limitation to all studies investigating the response of the peritoneal immune environment to endometriosis, including ours, is the relative low number of patient samples assayed. Albeit well characterized, our control population included women with other gynecologic diseases, which could conceivably impact the peritoneal microenvironment and immune cells, such as benign ovarian cysts or uterine fibroids. While it would be preferable to have a control group without any gynecological disorders, this is usually not feasible in this type of study that requires invasive surgery to collect samples that would not normally be performed on healthy individuals. In addition, given the heterogeneity in the stage of the disease and type and location of the lesions, larger sample sizes would be required to distinguish any differential effect of these disease characteristics on the peritoneal immune cell population. One way to overcome these limitations in the future would be to coordinate studies between different medical centers to enable studies based on larger cohorts.

To conclude, in this study, we have shown that changes in pMΦ polarization in endometriosis patients are limited to the CD14^+low^/CD68^+low^ pMΦ subpopulation, and that these changes are coupled with increases in T_h_2 and T_reg_ cells in a switch to a type 2 immune response. These findings resolve conflicting findings on the pMΦ response to endometriosis in the literature and show that changes are limited to this population. Future work should concentrate on characterizing the identity and origin of this subpopulation to better understand the pathogenesis of the disease, and on testing targeted experimental approaches, such as those described above, to correct the impaired immune response in endometriosis.

## Electronic Supplementary Material

ESM 1 (XLSX 10 kb)

ESM 2 Peritoneal macrophages in women with endometriosis are divided into CD14^+low^/CD68^+low^ and CD14^+high^/CD68^+high^ subpopulations that differ in the levels of the MΦ1 and MΦ2 subtypes. a Flow cytometry analysis of peritoneal monocytes/macrophages (pMO/pMΦ)reveals CD14^+low^ and CD14^+high^ subpopulations in women with endometriosis. Left: **a** representative contour plot of CD14 versus side scatter (SSC-A) analysis shows the low and high CD14 subpopulations. ITC – isotype control). Middle: the CD14^+high^ subpopulation is significantly more abundant than the CD14^+low^ subpopulation in women with endometriosis. Values are presented as a bee swarm dot plot analysis, with the mean values ± SD indicated. Statistical analysis was conducted using a multiple T-test with the Holm-Sidak method, using alpha = 0.05, for correction. Significant differences are indicated by the *p* value on the top of the graph. Analysis was conducted on pMΦ from *n* = 21 women with endometriosis. Right: a representative contour plot of CD68 versus CD14 signal with histograms of CD68 and CD14 signal shows two distinct subpopulations: a CD14^+low^/CD68^+low^ and a CD14^+high^/CD68^+high^ subpopulation (ITC- isotype control). **b** Flow cytometry analysis shows that relative abundance of the MΦ1 subtype is higher and the MΦ1 subtype lower in the pMΦCD14^+low/CD68+^ subpopulation, while relative abundance of the mixed MΦ1/MΦ2 subtype does not differ. Top: Representative scatter plots comparing CD163 and CD80 signal intensity in CD14^+low/CD68+^ (middle) and CD14^+high/CD68+^ (right) pMΦ subpopulations. Isotype control staining (ITC) is given on the left. Bottom: The CD14^low/CD68+^ pMΦ subpopulation has significantly higher levels of the M1 subtype and significantly lower levels of the M2 subtype compared to the CD14^+high^/CD68^+^ subpopulation, while levels of the mixed MΦ1/MΦ2 subtype do not significantly differ between the subpopulations. The data is presented as a bar graph with mean ± SD indicated. Statistical analysis was conducted using a two way ANOVA followed by the Holm-Sidak method for multiple comparison. Significant differences are indicated by the *p* value on the top of each graph, whereas non-significant differences are indicated by ns. Analysis was conducted on pMΦ from *n* = 21 women with endometriosis. (PPTX 125 kb)

ESM 3 The relative abundance of the MΦ2a and MΦ2b macrophage subtypes do not differ between the peritoneal macrophage CD14^+low/CD68+^ and CD14^+high/CD68+^ subpopulations of women with and without endometriosis. **a** Representative scatter plots showing CD86 and CD206 expression in the MΦ2 subtype (CD163^+^/CD80^−^) from the CD14^+low/CD68+^ (middle) and CD14^+high/CD68+^ (right) pMΦ subpopulations in women without (top) and women with (bottom) endometriosis. The scatter plots for isotype control staining (ITC) is given on the left of each group. **b** No significant difference between the CD14^+low/CD68+^ and CD14^+high/CD68+^ subpopulations was observed for the MΦ2a (CD163^+^/CD86^−^/CD206^+^), MΦ2a/b (CD163^+^/CD86^+^/CD206^+^) or the MΦ2b subtypes for women without (left) and with (right) endometriosis. Data is presented as bar graphs with the mean ± SD shown. Statistical analysis was conducted using two way ANOVA and the Holm-Sidak multiple comparison test. *p* > 0.05 indicates no significant difference between the groups. (PPTX 154 kb)
