# Supplementary material for: Endometriosis Patients Show an Increased M2 Response in the Peritoneal CD14+low/CD68+low Macrophage Subpopulation Coupled with an Increase in the T-helper 2 and T-regulatory Cells
Source: Reprod Sci. 2020 Jun 22;27(10):1920–31. doi: 10.1007/s43032-020-00211-9 (PMC7452931; doi:10.1007/s43032-020-00211-9)
Supplement: Supplementary file 2 — Peritoneal macrophages in women with endometriosis are divided into CD14+low/CD68+low and CD14+high/CD68+high subpopulations that differ in the levels of the MΦ1 and MΦ2 subtypes. a Flow cytometry analysis of peritoneal monocytes/macrophages (pMO/pMΦ)reveals CD14+low and CD14+high subpopulations in women with endometriosis. Left: a representative contour plot of CD14 versus side scatter (SSC-A) analysis shows the low and high CD14 subpopulations. ITC – isotype control). Middle: the CD14+high subpopulation is significantly more abundant than the CD14+low subpopulation in women with endometriosis. Values are presented as a bee swarm dot plot analysis, with the mean values ± SD indicated. Statistical analysis was conducted using a multiple T-test with the Holm-Sidak method, using alpha = 0.05, for correction. Significant differences are indicated by the p value on the top of the graph. Analysis was conducted on pMΦ from n = 21 women with endometriosis. Right: a representative contour plot of CD68 versus CD14 signal with histograms of CD68 and CD14 signal shows two distinct subpopulations: a CD14+low/CD68+low and a CD14+high/CD68+high subpopulation (ITC- isotype control). b Flow cytometry analysis shows that relative abundance of the MΦ1 subtype is higher and the MΦ1 subtype lower in the pMΦCD14+low/CD68+ subpopulation, while relative abundance of the mixed MΦ1/MΦ2 subtype does not differ. Top: Representative scatter plots comparing CD163 and CD80 signal intensity in CD14+low/CD68+ (middle) and CD14+high/CD68+ (right) pMΦ subpopulations. Isotype control staining (ITC) is given on the left. Bottom: The CD14low/CD68+ pMΦ subpopulation has significantly higher levels of the M1 subtype and significantly lower levels of the M2 subtype compared to the CD14+high/CD68+ subpopulation, while levels of the mixed MΦ1/MΦ2 subtype do not significantly differ between the subpopulations. The data is presented as a bar graph with mean ± SD indicated. Statistical analysis was conducted u [file 43032_2020_211_MOESM2_ESM.pptx]

## Slide 1
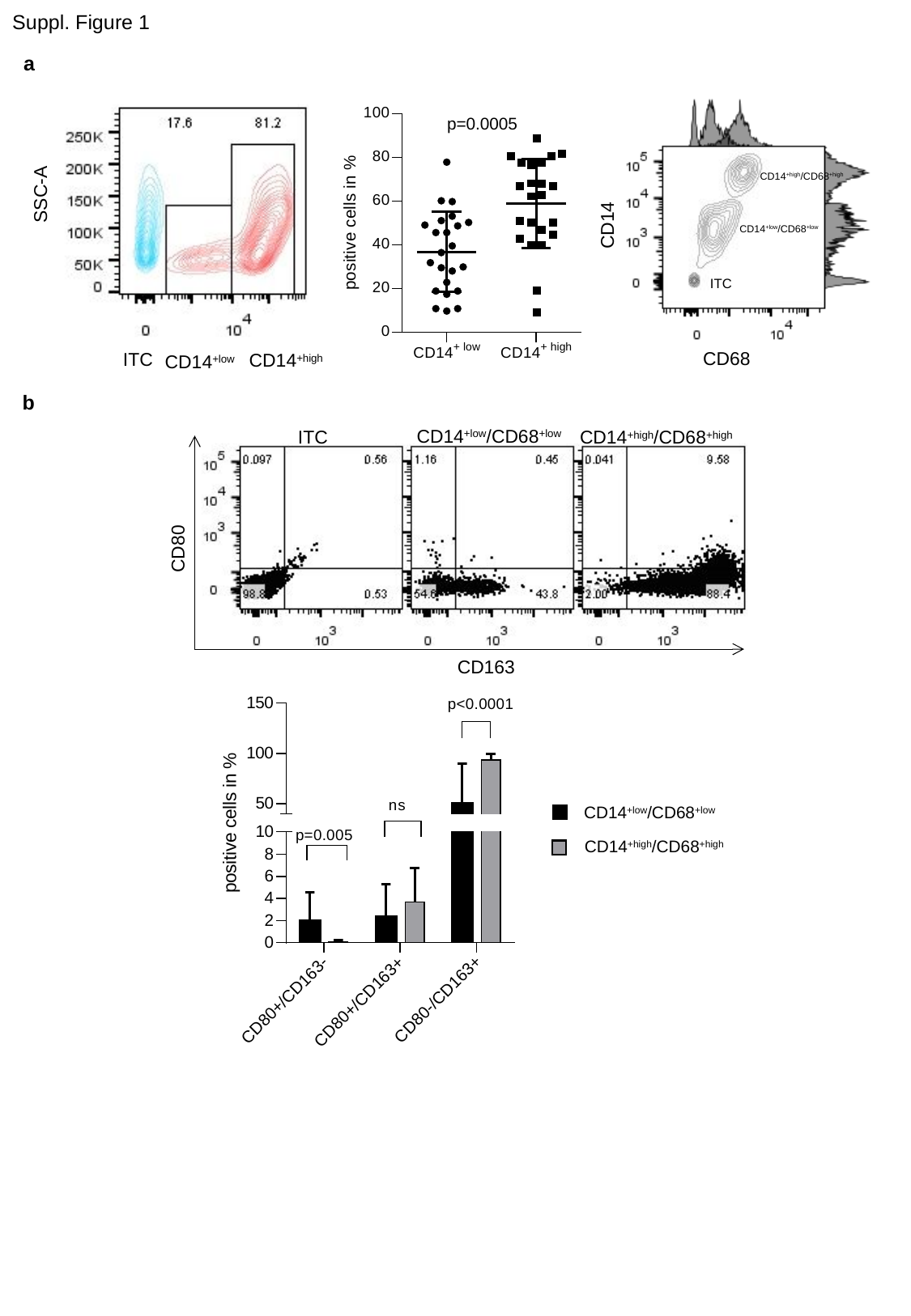

Suppl. Figure 1
a
SSC-A
ITC
CD14+low
CD14+high
p=0.0005
CD14+high/CD68+high
CD14
CD14+low/CD68+low
ITC
CD68
b
CD14+low/CD68+low
ITC
CD14+high/CD68+high
CD80
CD163
CD14+low/CD68+low
CD14+high/CD68+high
