# Supplementary material for: Endometriosis Patients Show an Increased M2 Response in the Peritoneal CD14+low/CD68+low Macrophage Subpopulation Coupled with an Increase in the T-helper 2 and T-regulatory Cells
Source: Reprod Sci. 2020 Jun 22;27(10):1920–31. doi: 10.1007/s43032-020-00211-9 (PMC7452931; doi:10.1007/s43032-020-00211-9)
Supplement: Supplementary file 3 — The relative abundance of the MΦ2a and MΦ2b macrophage subtypes do not differ between the peritoneal macrophage CD14+low/CD68+ and CD14+high/CD68+ subpopulations of women with and without endometriosis. a Representative scatter plots showing CD86 and CD206 expression in the MΦ2 subtype (CD163+/CD80−) from the CD14+low/CD68+ (middle) and CD14+high/CD68+ (right) pMΦ subpopulations in women without (top) and women with (bottom) endometriosis. The scatter plots for isotype control staining (ITC) is given on the left of each group. b No significant difference between the CD14+low/CD68+ and CD14+high/CD68+ subpopulations was observed for the MΦ2a (CD163+/CD86−/CD206+), MΦ2a/b (CD163+/CD86+/CD206+) or the MΦ2b subtypes for women without (left) and with (right) endometriosis. Data is presented as bar graphs with the mean ± SD shown. Statistical analysis was conducted using two way ANOVA and the Holm-Sidak multiple comparison test. p > 0.05 indicates no significant difference between the groups. (PPTX 154 kb) [file 43032_2020_211_MOESM3_ESM.pptx]

## Slide 1
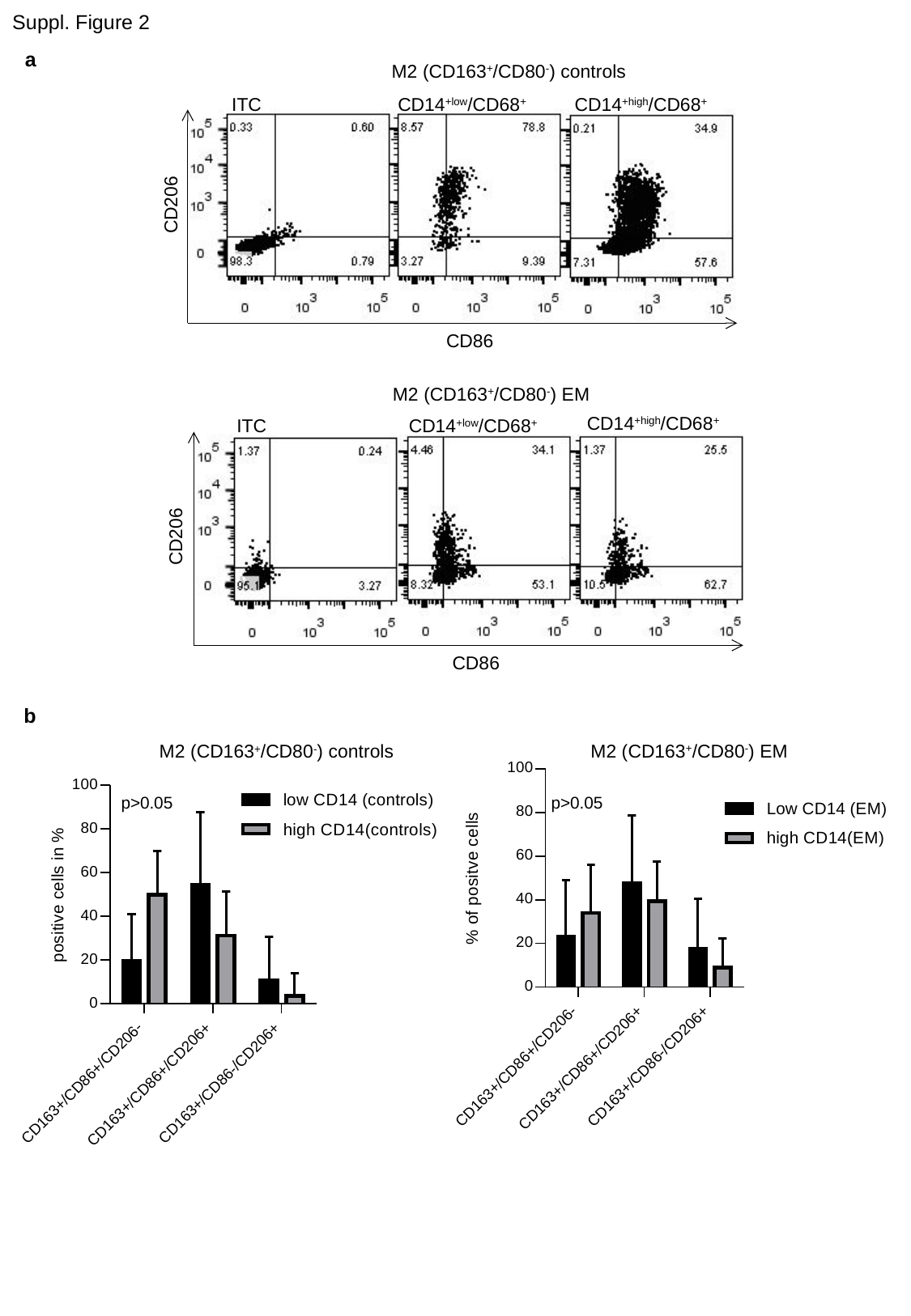

Suppl. Figure 2
a
M2 (CD163+/CD80-) controls
ITC
CD14+low/CD68+
CD14+high/CD68+
CD206
CD86
M2 (CD163+/CD80-) EM
CD14+high/CD68+
ITC
CD14+low/CD68+
CD206
CD86
b
M2 (CD163+/CD80-) EM
M2 (CD163+/CD80-) controls
p>0.05
p>0.05
